# Use of CFSE staining of borreliae in studies on the interaction between borreliae and human neutrophils

**DOI:** 10.1186/1471-2180-6-92

**Published:** 2006-10-18

**Authors:** Helena Tuominen-Gustafsson, Markus Penttinen, Jukka Hytönen, Matti K Viljanen

**Affiliations:** 1 Department of Medical Microbiology, University of Turku, Kiinamyllynkatu 13, FI-20520 Turku, Finland

## Abstract

**Background:**

Species of the tick-transmitted spirochete group *Borrelia burgdorferi *sensu lato (*B. burgdorferi*) cause Lyme borreliosis. Acute borrelial infection of the skin has unusual characteristics with only a mild local inflammatory response suggesting that the interaction between borreliae and the cells of the first-line defence might differ from that of other bacteria. It has been reported that human neutrophils phagocytose motile borreliae through an unconventional mechanism (tube phagocytosis) which is not observed with non-motile borreliae. Therefore, it would be of great interest to visualise the bacteria by a method not affecting motility and viability of borreliae to be able to study their interaction with the cells of the innate immunity. Carboxyfluorescein diacetate, succinimidyl ester (CFSE) labelling has been previously used for studying the adhesion of labelled bacteria to host cells and the uptake of labelled substrates by various cells using flow cytometry.

**Results:**

In this study, CFSE was shown to efficiently stain different genospecies of *B. burgdorferi *without affecting bacterial viability or motility. Use of CFSE staining allowed subsequent quantification of borreliae associated with human neutrophils with flow cytometry and confocal microscopy. As a result, no difference in association between different borrelial genospecies (*Borrelia burgdorferi *sensu stricto, *Borrelia afzelii*, *Borrelia garinii*), or between borreliae and the pyogenic bacterium *Streptococcus pyogenes*, with neutrophils could be detected. Borrelial virulence, on the other hand, affected association with neutrophils, with significantly higher association of a non-virulent mutant *B. burgdorferi *sensu stricto strain compared to the parental virulent wild type strain.

**Conclusion:**

These results suggest that the flow cytometric assay using CFSE labelled borreliae is a valuable tool in the analysis of the interaction between borreliae and human neutrophils. The results also indicate a clear difference in the association with neutrophils between virulent and non-virulent borrelial strains.

## Background

Species from the tick-transmitted spirochete group *Borrelia burgdorferi *sensu lato (*B. burgdorferi*) can cause Lyme borreliosis, which is a chronic multisystem inflammatory disorder [1]. It is still unclear whether clinical symptoms are caused by a chronic infection or whether the onset of the disease is triggered by borrelial antigens leading to the generation of an autoimmune disease. Although the exact mechanism of the pathogenesis remains to be elucidated, it is clear that acute borrelial infection has unusual characteristics. In most cases, only a mild local inflammatory response can be observed and occasionally the infection may be asymptomatic which is in striking contrast with many other bacteria causing skin infections (e.g. streptococci). These observations suggest that the interaction between borreliae and the first line defence might be severely altered. Therefore, it would be of great interest to be able to visualise the bacteria and to study their interaction with inflammatory cells involved in innate immunity. Furthermore, a reliable method would help to track the location of the bacteria during the first days of infection in animal models.

Borreliae are found only in their arthropod or mammalian hosts in the nature. They are spirochetal bacteria displaying a unique structure. Borreliae are 10–30 μm long, exhibit a planar waveform and are highly motile [2]. The motility differs from that of other bacteria as they are able to swim in a highly viscous medium, such as the connective tissue [3]. The bacteria demand a rich cultivation medium for growth in laboratory [4]. Changes in shape, loss of motility and death are the results of non-optimal growth conditions.

The shape and the motility of borreliae most likely affect their interactions (e.g. phagocytosis) with the cells of the immune system. Different mechanisms to phagocytose borreliae have been presented depending on different methodological approaches. Coiling phagocytosis was reported when imaging non motile borreliae [5], while a study using motile bacteria showed a novel "tube phagocytosis" [6]. These results indicate that studies should be performed using live motile borreliae to reveal in detail the mechanism(s) by which inflammatory cells phagocytose the bacteria *in vivo*.

The dye carboxyfluorescein diacetate, succinimidyl ester (CFSE) passively diffuses into cells. It is non-fluorescent until the acetate groups are cleaved by intracellular esterases to yield highly fluorescent and membrane non-permeable carboxyfluorescein succinimidyl ester, spontaneously and irreversibly coupling to cellular proteins by reaction with lysine side chains and other available amines [7]. The dye has been shown to be non-toxic enough to be widely used *in vivo *for visualising cells [7] or studying uptake of labelled substrates by the cells [8,9]. The label is inherited by daughter cells after cell division, with subsequent halving of fluorescence [10]. Also, labelling of bacteria has been performed with CFSE [11], and flow cytometric adherence assays with CFSE labelled bacteria have been used for the analysis of interactions between bacteria and eukaryotic cells [12-14].

Neutrophils are innate immune cells making up the first line of defence against invading bacteria. Studies on the association of borreliae with human neutrophils have mostly been performed using microscopic [15-17] or radioisotopic techniques [15,16]. Fluorescent labelling of borreliae would allow a much better visualisation of bacteria in microscopic studies, and also a means to quantitatively study the association between neutrophils and borreliae using flow cytometry. Indeed, a flow cytometric technique, staining *B. burgdorferi *with fluorescein-isothiocyanate (FITC), was described by Banfi and others in 1989 [18]. Flow cytometry has also been used to assess the uptake of PKH2-GL-labeled *B. burgdorferi *sensu stricto strain LW2 by dendritic cells [19]. However, thorough characterisation of the effect of labelling on borrelial viability has not been performed.

In this study, we wanted to develop a reliable, fast and easy quantification method for borrelial association with human neutrophils, with no effect on borrelial viability and motility. CFSE was chosen as the label because of its non-toxic and highly fluorescent nature and ability to stay fluorescent for several days. We report here the use of CFSE for staining different genospecies of *B. burgdorferi *and subsequent quantification of stained borreliae associated with neutrophils using flow cytometry and confocal microscopy. The method was found to be useful for comparing neutrophil association of different borrelial genotypes and of borreliae and the pyogenic bacterium *Streptococcus pyogenes*. Also, the effect of borrelial virulence was assessed with respect to association with human neutrophils.

## Results

### CFSE staining of Bb-HP, Ba and Bg

**Figure 1:**
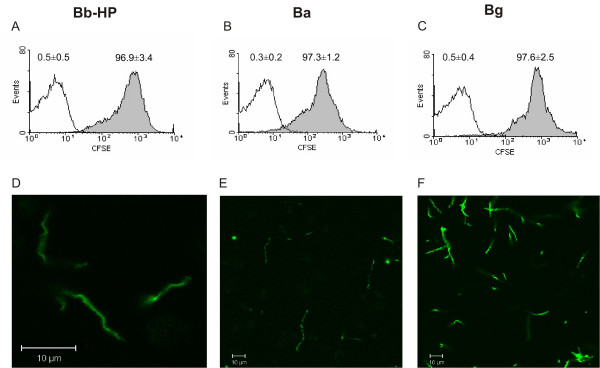
Staining of Bb-HP, Ba or Bg with CFSE. Panels (A)-(C) show histograms representative of three to eight different experiments. CFSE stained borreliae are shown as grey histograms. Borreliae treated according to the staining protocol with the exception of using PBS instead of CFSE served as negative control and are shown as white histograms. Percentages of fluorescent borreliae as mean ± standard deviation (SD) of three to eight different experiments are shown above the histograms. Panels (D)-(F) show microscopic images of CFSE-stained Bb-HP, Ba and Bg, respectively, and are representative of two to three experiments.

First we wanted to characterise the efficacy of CFSE to stain borreliae. As shown in Fig. 1A–C, a very high percentage of borreliae (96,9% of Bb-HP, 97,3% of Ba, and 97,6% of Bg) were stained with CFSE as determined by flow cytometry, while unstained bacteria showed only autofluorescence. These results were supported by confocal microscopy images showing a homogenous staining pattern of the bacteria (Fig. 1D–F).

**Figure 2:**
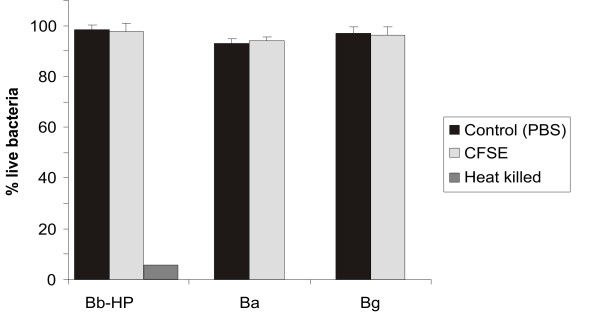
Viability of Bb-HP, Ba and Bg after staining with CFSE. Borreliae treated according to the staining protocol, with the exception of using PBS instead of CFSE, served as negative control. After the staining procedure, 3.2 μM propidium iodide (PI), staining the DNA of dead bacteria red, was added. Heat-killed and PI stained Bb-HP of a representative experiment are also shown to demonstrate the proper functioning of the PI-staining. The percent of PI-negative bacteria was determined by flow cytometry. Values are expressed as mean ± standard deviation from three to four different experiments. p ≥ 0.1 was obtained for each CFSE labelled strain versus corresponding control.

Borrelial viability after CFSE labelling was assessed using flow cytometric analysis of propidium iodide stained bacteria. No difference in the proportion of live bacteria between CFSE labelled and PBS treated borreliae was detected (Fig. 2). PI-staining of heat-killed borreliae was included in some experiments to demonstrate the proper functioning of the staining method. Thus, we conclude that CFSE labelling does not adversely affect borrelial viability.

### Association of CFSE-stained Bb-HP with human neutrophils

**Figure 3:**
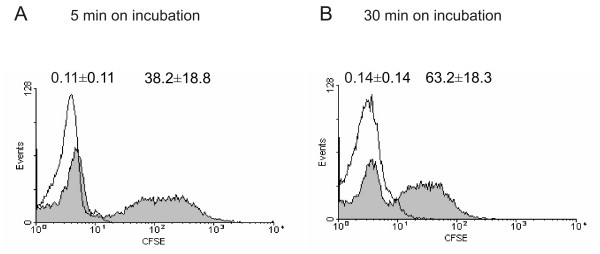
Association of CFSE-stained Bb-HP with human neutrophils. Neutrophils and CFSE-stained or unstained (PBS) Bb-HP were incubated for 5 (A) or 30 (B) min before the reaction was stopped by adding ice-cold PBS and samples were fixed. Flow cytometry was performed using "cell settings" showing only fluorescence associated with neutrophils. Panels (A) and (B) show histograms representative of five to eight different experiments carried out with neutrophils isolated from four to seven different donors. Association of CFSE stained borreliae with the cells is shown as grey histograms and of unstained control bacteria as white histograms. Percentages of fluorescent neutrophils are shown above the histograms as mean ± standard deviation (SD) of five to eight different experiments carried out with cells from four to seven different donors.

When neutrophils were incubated with CFSE-stained Bb-HP, fluorescence could be detected cytometrically using cell setting parameters detecting only fluorescence arising from the labelled bacteria associated with neutrophils. Neutrophils were divided into two separate populations, one without CFSE fluorescence (only autofluorescence) and another with detectable CFSE fluorescence. The percent of cells in each population was dependent on the incubation time of neutrophils and CFSE-stained Bb-HP, with more CFSE fluorescent neutrophils as a function of incubation time (Fig. 3). Results with neutrophils incubated with CFSE-stained Ba or Bg were similar to the data obtained with Bb-HP (data not shown).

### Localisation of CFSE-stained Bb-HP incubated with human neutrophils

**Figure 4:**
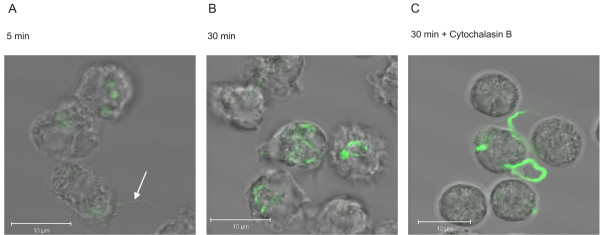
Localisation of CFSE-stained Bb-HP incubated with human neutrophils. Confocal microscopy was used to demonstrate intracellular localisation of CFSE-stained Bb-HP. Neutrophils and CFSE-stained Bb-Hp were incubated for 5 min (A) or 30 min (B) before the reaction was stopped by adding ice-cold PBS and samples were fixed. The white arrow on panel (A) indicates a borrelia bacterium attached to neutrophil surface. In panel (C), neutrophils were pre-treated for 10 min with 5 μg/ml Cytochalasin B before addition of CFSE-stained Bb-HP for 30 min. Images shown are "stack images" taken at a defined cross section of the neutrophil, as close to the centre of the cell as possible. The white bar represents 10 μm. Images are representative of two to three samples carried out with neutrophils from two to three different donors.

To be able to localise CFSE-stained Bb-HP associated with neutrophils, some experiments were performed using confocal microscopy of fixed samples. After 5 min of incubation, some of the neutrophil-associated CFSE-stained Bb-HP were bound to the plasma membrane of the cells (Fig. 4A). After 30 min of incubation, when looking at the localisation of fluorescent material at different cross-sections of the neutrophils, most fluorescence appeared to be inside the cells (Fig. 4B). The fluorescent material inside the cell was appearing in a "spotted" fashion, and intact Bb-HP could not be detected, suggesting localization of fluorescence in phagosomes. After pre-treatment of neutrophils with Cytochalasin B, inhibiting phagocytosis, a dramatic inhibition of intracellular localisation could be detected (Fig. 4C). Similar data were obtained with neutrophils incubated with CFSE-stained Ba or Bg (data not shown).

### Kinetics of association of CFSE-stained Bb-HP, Ba and Bg with human neutrophils

**Figure 5:**
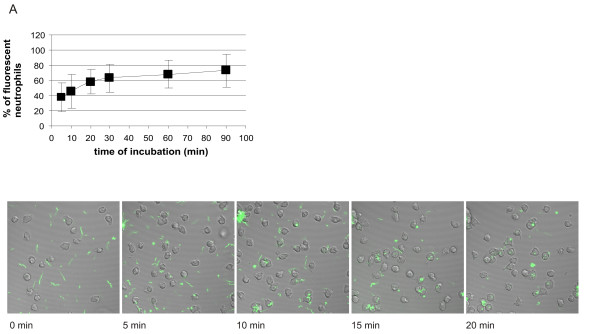
Kinetics of association of CFSE-stained Bb-HP, Ba or Bg with human neutrophils. Neutrophils and CFSE-stained Bb-Hp (Fig. 5), Ba (Fig. 6) or Bg (Fig. 7) were incubated for 5, 10, 20, 30, 60 or 90 min before the reaction was stopped by adding ice-cold PBS and samples were fixed. Flow cytometry was performed using "cell settings" showing only fluorescence associated with neutrophils. Values are expressed as mean ± standard deviation and are obtained from seven to nine different experiments carried out with neutrophils isolated from six to eight different donors. Confocal microscopy was performed using live neutrophils and CFSE-stained Bb-HP (Fig. 5), Ba (Fig. 6) or Bg (Fig. 7). Scanning was performed at 37°C and images were stored at intervals of 2.5 seconds. Pictures show images taken at 0 (immediately after addition of bacteria into the culture dish mounted in the microscope), 5, 10, 15, and 20 min after addition of fluorescent bacteria. The tendency of Bg to form aggregates can be seen in the pictures. Images are representative of three to four "live-sessions" carried out with neutrophils from two to three different donors.

**Figure 6:**
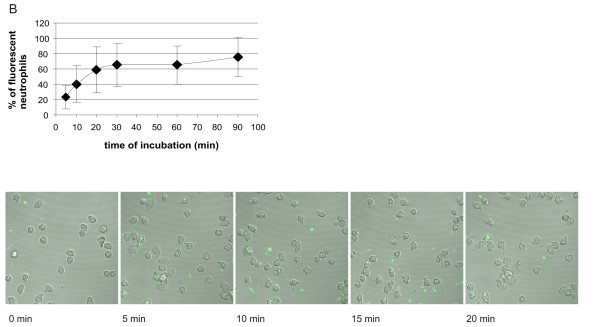
Kinetics of association of CFSE-stained Bb-HP, Ba or Bg with human neutrophils. Neutrophils and CFSE-stained Bb-Hp (Fig. 5), Ba (Fig. 6) or Bg (Fig. 7) were incubated for 5, 10, 20, 30, 60 or 90 min before the reaction was stopped by adding ice-cold PBS and samples were fixed. Flow cytometry was performed using "cell settings" showing only fluorescence associated with neutrophils. Values are expressed as mean ± standard deviation and are obtained from seven to nine different experiments carried out with neutrophils isolated from six to eight different donors. Confocal microscopy was performed using live neutrophils and CFSE-stained Bb-HP (Fig. 5), Ba (Fig. 6) or Bg (Fig. 7). Scanning was performed at 37°C and images were stored at intervals of 2.5 seconds. Pictures show images taken at 0 (immediately after addition of bacteria into the culture dish mounted in the microscope), 5, 10, 15, and 20 min after addition of fluorescent bacteria. The tendency of Bg to form aggregates can be seen in the pictures. Images are representative of three to four "live-sessions" carried out with neutrophils from two to three different donors.

**Figure 7:**
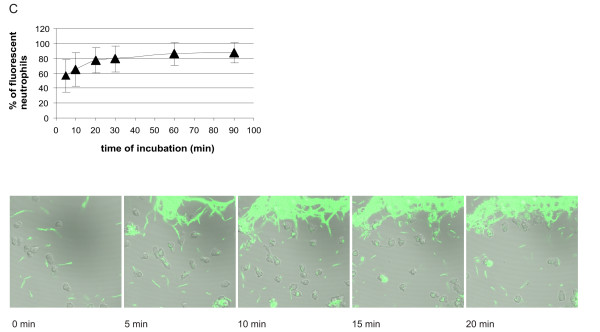
Kinetics of association of CFSE-stained Bb-HP, Ba or Bg with human neutrophils. Neutrophils and CFSE-stained Bb-Hp (Fig. 5), Ba (Fig. 6) or Bg (Fig. 7) were incubated for 5, 10, 20, 30, 60 or 90 min before the reaction was stopped by adding ice-cold PBS and samples were fixed. Flow cytometry was performed using "cell settings" showing only fluorescence associated with neutrophils. Values are expressed as mean ± standard deviation and are obtained from seven to nine different experiments carried out with neutrophils isolated from six to eight different donors. Confocal microscopy was performed using live neutrophils and CFSE-stained Bb-HP (Fig. 5), Ba (Fig. 6) or Bg (Fig. 7). Scanning was performed at 37°C and images were stored at intervals of 2.5 seconds. Pictures show images taken at 0 (immediately after addition of bacteria into the culture dish mounted in the microscope), 5, 10, 15, and 20 min after addition of fluorescent bacteria. The tendency of Bg to form aggregates can be seen in the pictures. Images are representative of three to four "live-sessions" carried out with neutrophils from two to three different donors.

The kinetics of association of all three tested genospecies of *B. burgdorferi *with human neutrophils was the same (Figs 5, 6, 7). It was biphasic with a faster first phase lasting for about 20 min, followed by a much slower second phase almost reaching plateau levels at 30 min and lasting for at least 90 min. The results were supported by those performed in parallel using live imaging of cells by confocal microscopy. At 5 min of incubation about 20–60% of neutrophils were associated with fluorescent borreliae, and the maximal amount of neutrophils associated with fluorescent borreliae was about 60–80%. A somewhat higher percentage of neutrophils seemed to be consistently associated with Bg compared to the other two strains, but because of variation in the phagocytic activity of neutrophils from different donors, no significant difference occurred between the tested genospecies.

**Figure 8:**
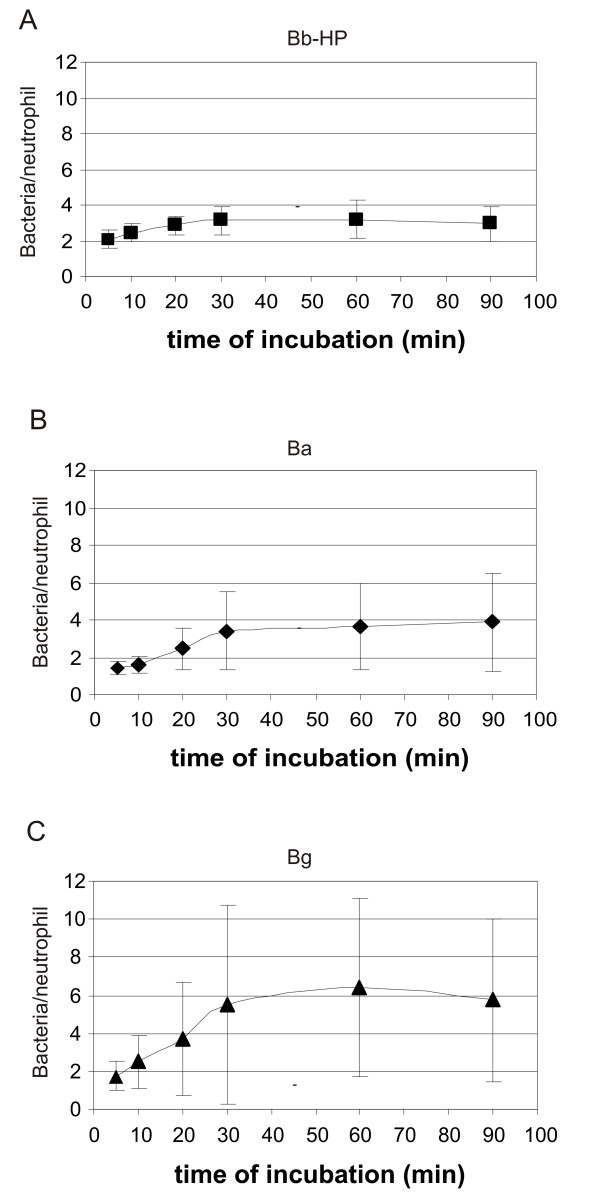
The amount of borreliae associated with neutrophils at different times of incubation. (A) Bb-HP, (B) Ba, (C) Bg. The mean fluorescence intensity (MFI) value associated with neutrophils (cell setting) was divided with the MFI for Bb-HP, Ba or Bg (bacterial settings) in each experiment. Values are expressed as mean ± standard deviation and are obtained from five to six different experiments carried out with neutrophils from four to five different donors.

The estimation of number of bacteria associated with a single neutrophil was based on MFI values as described in the methods section. The amount of fluorescent material associated with a single neutrophil was biphasic, with a faster first phase lasting for about 30 min, followed by a plateau level with no further increase in associated fluorescent material (Fig. 8). The kinetic profile was the same for the three tested borrelial strains. The kinetics of the bacteria/PMN curves (Fig. 8) paralleled those reflecting the amount of neutrophils involved (Figs 5, 6, 7).

### Effect of virulence on association of B31 with human neutrophils

**Figure 9:**
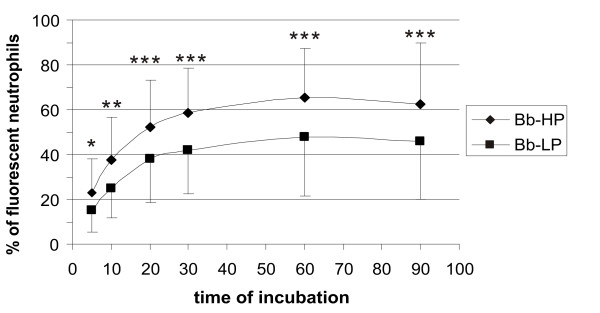
Effect of virulence on association of *B. burgdorferi *with human neutrophils. The non-virulent Bb-HP strain was compared to the virulent Bb-LP strain with respect to kinetics of association with human neutrophils. Values are expressed as mean ± standard deviation and obtained from four different experiments carried out in duplicates with neutrophils isolated from four different donors. Asterisks express statistical significance between the two strains: * p ≤ 0.05, ** p ≤ 0.01, *** min p ≤ 0.001.

To investigate the role of borrelial virulence in association of borreliae with human neutrophils, we compared the high and low passage strains of *B. burgdorferi *ss B31 (Bb-HP and Bb-LP). Bb-HP strain lacks several plasmids including the linear virulence plasmid 25 (lp25) as determined by PCR (data not shown), and is unable to cause infection in borrelia-sensitive C3H/He mice (data not shown). Bb-LP is a low passage strain (less that 12 passages) containing lp25 and tested to be virulent in C3H/He mice (data not shown). As a result, we found that the association of Bb-HP with human neutrophils was significantly higher than the association of Bb-LP at all tested time points (Fig. 9).

### Association of neutrophils with *Streptococcus pyogenes*

**Figure 10:**
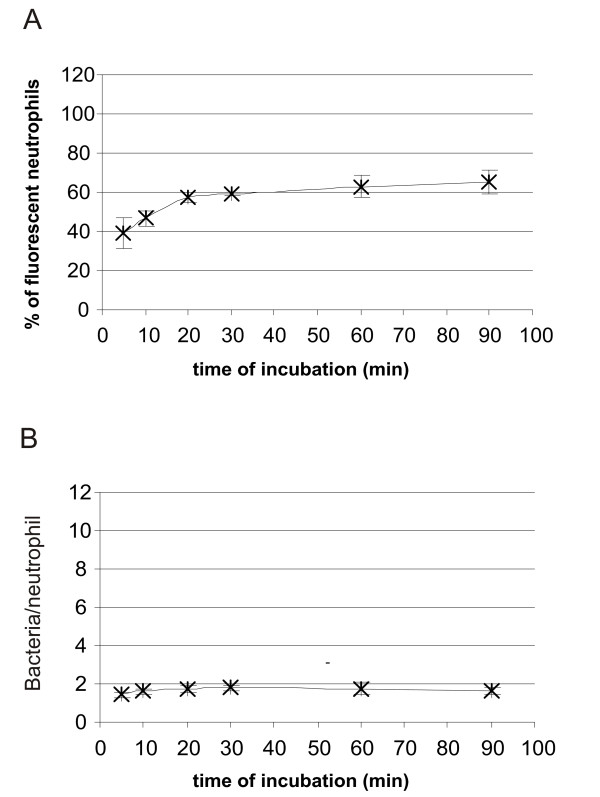
(A) Kinetics of association of CFSE-stained *S. pyogenes *by human neutrophils. Neutrophils and CFSE-stained *S. pyogenes *were incubated for 5, 10, 20, 30, 60 or 90 min before the reaction was stopped by adding ice-cold PBS and samples were fixed. Flow cytometry was performed using "cell settings" showing only fluorescence associated with neutrophils. Values are expressed as mean ± standard deviation and are obtained from three different experiments carried out with neutrophils isolated from three different donors. (B) The amount of *S. pyogenes *associated with neutrophils at different timepoints of incubation. The mean fluorescence intensity (MFI) value associated with neutrophils (cell setting) was divided with the MFI for *S. pyogenes *(bacterial settings) in each experiment. Values are expressed as mean ± standard deviation and are obtained from three different experiments carried out with neutrophils from three different donors.

When incubating neutrophils with *S. pyogenes *a biphasic association was registered with respect to percentage of neutrophils involved (Fig. 10A). This association curve closely resembled that of neutrophils and borreliae (Figs 5, 6, 7). The biphasic shape of the curve was not so prominent as with borreliae, and a plateau level was reached almost immediately in the amount of fluorescent *S. pyogenes *associated per neutrophil (Fig. 10B).

## Discussion

Different methods are available for quantifying phagocytosis of bacteria by human neutrophils [20]. One advantage with using labelled bacteria is the simplicity of the assay. Flow cytometry is rapid, which allows testing of many time-points or different experimental settings in one experiment. Furthermore, flow cytometry is sensitive and requires small sample volumes. A much larger amount of cells can be quantitated compared to microscopy. Flow cytometry is not as prone to errors because of individual interpretation by the operator as microscopy. On the other hand, microscopic techniques have the advantage that they allow direct visual assessment of the interaction between bacteria and cells.

CFSE has been reported non-toxic enough to be used *in vivo*. The atoxic nature of the dye is supported by the findings of this study. CFSE did neither adversely affect borrelial growth (data not shown) nor motility. The fluorescence of CFSE is detectable up to ten cycles of cell division both *in vivo *and *in vitro *[21]. *In vitro*, the fluorescence of CFSE labelled borreliae in cultivation lasted for six days (data not shown), which may allow tracing of CFSE-labelled borreliae in eukaryotic cells *in vivo*. The uptake of CFSE-labelled borreliae by different cell types can be followed up on a day to day basis to get information about location of borreliae in early infection. These findings show that CFSE offers marked advantages compared to other labels previously used to stain *B. burgdorferi *[18]. We also performed CFSE-staining experiments in the presence of 0.2% BSA, which is in many laboratories added to PBS-buffer to protect the outer membrane of borreliae, but under these conditions the bacteria were not labelled at all (data not shown).

One major concern in phagocytosis assays is to distinguish actually internalised bacteria from those only attached to the cell. In this study, microscopic data showed that some fluorescent bacteria were attached to the cell membrane at early time points and attachment could not be distinguished from phagocytosis. Thus, the results should be interpreted as association of the bacteria and the cells, including both attachment and uptake. Several methods have been used to differentiate attachment from uptake [22-24]. One approach is based on the quenching effect of trypan blue on extracellular fluorescence. However, the quenching effect differs significantly depending on the methodological approach [22,24]. Ethidium bromide (EB), previously used to change the emission spectrum of FITC from green to red [25], also affected the autofluorescence of non-labelled particles by causing red unspecific background fluorescence (data not shown). As modulation of extracellular fluorescence might affect fluorescence coming from inside the cells, we did not use any quencher. Further studies are needed to distinguish attachment and phagocytosis in this setup.

To our knowledge, comparative studies of phagocytosis of *B. burgdorferi*, *B. afzelii *and *B. garinii *by human blood leukocytes have not been done. In one study, no significant differences in uptake of radio-labelled *B. burgdorferi *IRS, *B. garinii *PBi and *B. afzelii *VS461 by hepatic macrophages were observed when perfusing rat livers [26]. Our results are in accordance with this finding indicating similar association of the different genospecies with human neutrophils.

Passaging of borreliae may lead to plasmid losses and subsequent changes in the structure and infectivity of borreliae [27]. *B. burgdorferi *organisms lacking lp25 are completely non-infectious [28]. Our results suggest that plasmid content and infectivity of borreliae significantly affect the rate of association of the bacteria with human neutrophils, as association of non-virulent Bb-HP with human neutrophils was significantly higher at all tested time points. Previously, contradictory results have been presented regarding the infectivity of *B. burgdorferi *versus resistance to phagocytosis by leukocytes. Georgilis et al. [29] stated, based on microscopic evaluation, that highly infective, low-passage strains of *B. burgdorferi *(N40 and 297) resist phagocytosis by human blood phagocytes much better than high-passage variants of the same strains with low infectivity. On the other hand, Al-Robaiy and others have shown that passaging and infectivity do not significantly affect internalization rates of two strains of *B. burgdorferi *(B31 and N40) by neutrophils based on flow cytometric studies [24]. In their studies, whole blood was used instead of isolated neutrophils. Our premilinary results show that the Bb-HP mutant also lacks other plasmids than the virulence plasmid lp25. Thus, it is possible that the difference in interaction of this strain with neutrophils is affected by a plasmid or plasmids other than lp25, which will be tested in the future.

We also wanted to use the method for comparing the phagocytosis and kinetics of association of borreliae and a pyogenic bacterium to human neutrophils *in vitro*. *S. pyogenes *causes skin infections ranging from superficial impetigo to life-threatening nectrotizing fasciitis of soft tissues characterised by a large number of infiltrating neutrophils [30]. In contrast, infiltrates of erythema migrans skin lesions caused by *B. burgdorferi *have been reported to contain only very few neutrophils [31]. Unexpectedly, no difference in the percentage of neutrophils involved in the interaction with borreliae or *S. pyogenes*, or in the kinetics of the association could be registered. It seems that neutrophils *in vitro *respond to borreliae more efficiently than *in vivo*. Factors in tick saliva inhibiting the functions of neutrophils [32], or different expression of borrelial proteins *in vivo *and *in vitro *affecting the interaction between borreliae and immune cells [33] might offer an explanation.

By dividing the mean fluorescence intensity (MFI) value associated with neutrophils (cell settings) with the MFI for Bb-HP, Ba, Bg or *S. pyogenes *(bacterial settings) in each experiment, we got an estimation about the amount of fluorescent material associated with one neutrophil at each time point tested. These values cannot, however, be directly interpreted as the number of bacteria associated per neutrophil. The MFI values of the three genospecies of borreliae and of *S. pyogenes *varied between the different experiments. This was, at least partly, due to some variation in the length of the spirochetes, as assessed by phase-contrast microscopy (data not shown), but also to the fact that clusters of borreliae could appear. Especially, *B. garinii *showed extensive clustering, which may explain the great variation in the amount of bacterial material associated with neutrophils in experiments with this genospecies. However, kinetic data could be obtained showing that more fluorescent material was associated with neutrophils in the first 30 min, regardless of the exact number of bacteria involved. No difference in kinetics was observed between the three borrelial genospecies, while studies with *S. pyogenes *suggest that neutrophils became saturated with this bacterium more rapidly than with borreliae. The kinetic data is supported by microscopic findings showing that each neutrophil could bind several borreliae, and that the number of borreliae per cell increased during the first 20 minutes.

## Conclusion

This study shows that CFSE efficiently labels the three genospecies of borreliae without affecting viability of the bacteria. CFSE labelling of borreliae can be used for quantitative analysis of borrelial association with human neutrophils using flow cytometry. The method offers a fast way to analyse a large number of cells in different settings in one experiment and provides kinetic data on association of borreliae and neutrophils. Also, the method allows the comparison of different borrelial strains, and the impact on phagocytosis of borrelial qualities such as infectivity can be assessed.

## Methods

### Bacterial strains and culture conditions

The following borrelial strains were used: *B. burgdorferi *sensu stricto B31 (ATCC 35210) high passage (Bb-HP) and B31 low passage (Bb-LP) (gifts from S. Bergström, Umeå, Sweden), *B. afzelii *B023 (Ba) isolated from a skin biopsy specimen of a German patient, high passage (a gift from S. Batsford, Freiburg, Germany), and *B. garinii *Å218/98 (Bg), a Finnish tick isolate infective in mice, low-passage.

Borreliae were maintained in liquid Barbour-Stoenner-Kelly II (BSK II) medium at 34°C and passaged weekly. Low passage strains, Bb-LP and Bg, were passaged five to twelve times. Plasmid content of the Bb-LP and Bb-HP strains was assessed by PCR. DNA from bacteria was isolated with a commercial nucleic acid extraction kit (Wizard Genomic, DNA Purification Kit, Promega Corporation, Madison, USA). Amplification and detection of the 12 linear and 9 circular plasmids were performed as described by Elias et al[34]. Bb-LP was monitored routinely on a monthly basis to assure that there was no loss of plasmids.

*S. pyogenes *NZ131 (a gift from M. Chaussee, Hamilton, Montana, USA) was grown on Todd Hewitt plates or broth (Becton Dickinson Microbiology Systems, MD, USA) supplemented with 0.5% yeast extract (IDG, Lancashire, UK) over night at 37°C. All bacteria were stored at -70°C in growth medium containing 15% glycerol.

### Staining of bacteria with CFSE

Bb-HP, Bb-LP, Ba, Bg or *S. pyogenes *were washed once in phosphate buffered saline (PBS) and diluted in 500 μl of PBS. 500 μl of PBS containing 10 μM CFSE (Molecular Probes, Eugene, Oregon, USA) was added to the bacterial suspension (final volume 1 ml and final concentration of CFSE 5 μM) and subsequently incubated for 10 min at room temperature and protected from light. The bacterial suspension was washed twice in PBS supplemented with 5% fetal calf serum (FCS; Hyclone, Utah, USA). Borreliae were counted in a Neubauer counting chamber and suspended in Hanks' balanced salt solution (HBSS) supplemented with 0.25% bovine serum albumin (BSA; Wilfrid Smith, Edgware, UK) at a concentration of 1.5 × 10^8 ^bacteria/ml. The amount of *S. pyogenes *colony forming units (CFU) was determined spectrofotometrically by determining OD 600, and calculation of CFU concentration was based on the finding that OD 0.3 equals to 10^8 ^CFU/ml. *S. pyogenes *was suspended in HBSS-0,25% BSA at a concentration of 2.5 × 10^8 ^CFU/ml.

### The assessment of borrelial viability after staining with CFSE

To study the viability and the motility of borreliae, Bb-HP, Ba and Bg were stained with 5 μM CFSE. As a control, the same amount of Bb-HP, Ba and Bg were treated in the same way with the exception of using PBS instead of CFSE. An aliquot of CFSE treated or control borreliae were stained with 3.2 μM propidium iodide (PI; Molecular Probes), which stains the DNA of dead cells red, and analysed by flow cytometry. Also, to demonstrate that the PI-staining works properly, heat-killed (45 min at 56°C) borreliae were analysed.

### Isolation of neutrophils

Human neutrophils were isolated from heparin-anticoagulated venous blood of healthy volunteers as previously described [35]. Briefly, Dextran T-500 (Pharmacia Biotech, Uppsala, Sweden) sedimentation and Percoll (Pharmacia Biotech) centrifugation were used, remaining erythrocytes were lysed with 0.83% ammonium chloride, and neutrophils were washed with PBS. The neutrophils were counted in a Neubauer counting chamber and suspended in HBSS-0.25% BSA at a concentration of 1 × 10^7^/ml.

### Flow cytometric analyses

Bb-HP, Bb-LP, Ba, Bg or *S. pyogenes *were stained with CFSE as described above. The borrelial suspensions were fixed by adding PBS containing 1% formalin when the fluorescence on the bacteria alone was analysed. Unstained Bb-HP, Ba or Bg served as negative controls.

In the phagocytosis assay, 3 × 10^6 ^CFSE labelled borreliae or 5 × 10^6 ^CFU of CFSE labelled *S. pyogenes *in PBS were added to a suspension of 0.5 × 10^6 ^neutrophils diluted in HBSS-0.25% BSA (borrelia to neutrophil ratio 6:1; *S. pyogenes *CFU to neutrophil ratio 10:1). Normal human serum (NHS) was prepared by pooling serum from six healthy donors as previously described [36]. 10% pooled NHS was added as a source of complement. The final volume of the incubation mixture was 500 μl. Incubation took place protected from light at 37°C keeping the test tubes on a shaker, 45 rpm. The interactions of bacteria and cells were stopped at indicated time points by washing with ice-cold PBS. Finally, the samples were fixed by adding PBS containing 1% formalin. Neutrophils incubated with unstained bacteria served as negative controls.

FACS analyses were done by using a FACScalibur flow cytometer (Beckton-Dickinson, San Jose, USA) and CellQuest (Beckton-Dickinson) software. Routinely, 10, 000 cells were collected for analyses. Different parameters were selected when analysing suspensions containing only bacteria ("bacterial settings") and suspensions containing neutrophils incubated with bacteria ("cell settings"). Appropriate gates were selected to exclude the autofluorescence arising from the bacteria or the cells through analysing samples of non-labelled borreliae and neutrophils without labelled bacteria, respectively. Thus, when labelled bacteria were incubated with neutrophils, fluorescence from bacteria associated with the cells could be detected using "cell settings". The few contaminating mononuclear cells and erythrocytes in the neutrophil samples were excluded during the analyses based on the scatter parameters.

Mean fluorescence intensity (MFI) values correlate to the average amount of fluorescent material associated with a single cell. By dividing the MFI of neutrophils obtained with "cell settings" (only cells with associated bacteria) with the MFI for Bb-HP, Ba, Bg or *S. pyogenes *(bacterial settings) in each experiment, an estimation on the amount of bacterial material associated with one neutrophil at each time point was achieved.

### Confocal microscopy

Bb-HP, Ba, and Bg were stained with CFSE as described above. An aliquot of neutrophils was treated with 5 μg/ml Cytochalasin B on a shaker (45 rpm) for 10 min at 37°C. 3 × 10^6 ^borreliae were added to a suspension of 0.5 × 10^6 ^neutrophils (± Cytochalasin B) diluted in HBSS-0.25% BSA (borrelia to cell ratio 6:1). 10% pooled NHS was added as a source of complement. The final volume of the incubation mixture was 500 μl. Incubation took place protected from light for 5 minutes (only Cytochalasin B untreated neutrophils) or for 30 minutes (both Cytochalasin B treated and untreated neutrophils) at 37°C keeping the test tubes on a shaker (45 rpm). The interactions of bacteria and cells were stopped by washing with ice-cold PBS. Finally, the samples were fixed by adding PBS containing 1% formalin. Samples were transferred to slides and scanned under a 63 × objective using a Zeiss LSM 510 META confocal microscope (Oberkochen, Germany) equipped with an argon-krypton laser. "Stack images" were taken at different cross sections of the neutrophils perpendicularly to the bottom of the culture dish.

Alternatively, live-imaging of cells and bacteria was performed. Neutrophils were kept in an incubator at 37°C prior to experiments. 0.5 × 10^6 ^neutrophils diluted in HBSS-0.25% BSA were transferred to 35 mm glass bottom culture dishes (MatTek CORPORATION, Ashland, USA). Pooled NHS was added to give a final concentration of 10% and scanning was performed at 37°C using the confocal microscope. After addition of 3 × 10^6 ^CFSE-labelled Bb-HP, Ba or Bg into the culture dish already mounted in the microscope (final volume 500 μl), imaging was started. Images were stored at intervals of 2.5 seconds and imaging was continued for 20 min.

### Statistics

Student's two-tailed paired *t*-test was used for determining the statistical significance.

## List of abbreviations

CFSE, carboxyfluorescein diacetate, succinimidyl ester

Bb-HP,* Borrelia burgdorferi *sensu stricto, high passage

Bb-LP, *Borrelia burgdorferi *sensu stricto, low passage

Ba,* Borrelia afzelii*

Bg, *Borrelia garinii*.

## Authors' contributions

HT-G had the primary responsibility of the planning of the study and carried out the experiments, MP contributed to the planning of the study, JH contributed to the planning of the study and to the writing of the manuscript, and MKV directed the design and execution of the project. All authors have read and approved the manuscript.

## References

[B1] Steere AC (2001). Lyme disease. N Engl J Med.

[B2] Goldstein SF, Charon NW, Kreiling JA (1994). Borrelia burgdorferi swims with a planar waveform similar to that of eukaryotic flagella. Proc Natl Acad Sci U S A.

[B3] Kimsey RB, Spielman A (1990). Motility of Lyme disease spirochetes in fluids as viscous as the extracellular matrix. J Infect Dis.

[B4] Barbour AG (1984). Isolation and cultivation of Lyme disease spirochetes. Yale J Biol Med.

[B5] Rittig MG, Krause A, Haupl T, Schaible UE, Modolell M, Kramer MD, Lutjen-Drecoll E, Simon MM, Burmester GR (1992). Coiling phagocytosis is the preferential phagocytic mechanism for Borrelia burgdorferi. Infect Immun.

[B6] Suhonen J, Hartiala K, Viljanen MK (1998). Tube phagocytosis, a novel way for neutrophils to Phagocytize borrelia burgdorferi. Infect Immun.

[B7] Weston SA, Parish CR (1990). New fluorescent dyes for lymphocyte migration studies. Analysis by flow cytometry and fluorescence microscopy. J Immunol Methods.

[B8] Iyoda T, Shimoyama S, Liu K, Omatsu Y, Akiyama Y, Maeda Y, Takahara K, Steinman RM, Inaba K (2002). The CD8+ dendritic cell subset selectively endocytoses dying cells in culture and in vivo. J Exp Med.

[B9] Kulprathipanja NV, Kruse CA (2004). Microglia phagocytose alloreactive CTL-damaged 9L gliosarcoma cells. J Neuroimmunol.

[B10] Lyons AB, Parish CR (1994). Determination of lymphocyte division by flow cytometry. J Immunol Methods.

[B11] Hoefel D, Grooby WL, Monis PT, Andrews S, Saint CP (2003). A comparative study of carboxyfluorescein diacetate and carboxyfluorescein diacetate succinimidyl ester as indicators of bacterial activity. J Microbiol Methods.

[B12] Sethman CR, Doyle RJ, Cowan MM (2002). Flow cytometric evaluation of adhesion of Streptococcus pyogenes to epithelial cells. J Microbiol Methods.

[B13] Logan RP, Robins A, Turner GA, Cockayne A, Borriello SP, Hawkey CJ (1998). A novel flow cytometric assay for quantitating adherence of Helicobacter pylori to gastric epithelial cells. J Immunol Methods.

[B14] Hytönen J, Haataja S, Finne J (2006). Use of flow cytometry for the adhesion analysis of Streptococcus pyogenes mutant strains to epithelial cells: investigation of the possible role of surface pullulanase and cysteine protease, and the transcriptional regulator Rgg. BMC Microbiol.

[B15] Benach JL, Fleit HB, Habicht GS, Coleman JL, Bosler EM, Lane BP (1984). Interactions of phagocytes with the Lyme disease spirochete: role of the Fc receptor. J Infect Dis.

[B16] Szczepanski A, Furie MB, Benach JL, Lane BP, Fleit HB (1990). Interaction between Borrelia burgdorferi and endothelium in vitro. J Clin Invest.

[B17] Peterson PK, Clawson CC, Lee DA, Garlich DJ, Quie PG, Johnson RC (1984). Human phagocyte interactions with the Lyme disease spirochete. Infect Immun.

[B18] Banfi E, Cinco M, Perticarari S, Presani G (1989). Rapid flow cytometric studies of Borrelia burgdorferi phagocytosis by human polymorphonuclear leukocytes. J Appl Bacteriol.

[B19] Filgueira L, Nestle FO, Rittig M, Joller HI, Groscurth P (1996). Human dendritic cells phagocytose and process Borrelia burgdorferi. J Immunol.

[B20] Hampton MB, Winterbourn CC (1999). Methods for quantifying phagocytosis and bacterial killing by human neutrophils. J Immunol Methods.

[B21] Lyons AB (2000). Analysing cell division in vivo and in vitro using flow cytometric measurement of CFSE dye dilution. J Immunol Methods.

[B22] Hed J, Hallden G, Johansson SG, Larsson P (1987). The use of fluorescence quenching in flow cytofluorometry to measure the attachment and ingestion phases in phagocytosis in peripheral blood without prior cell separation. J Immunol Methods.

[B23] Tabrizi SN, Robins-Browne RM (1993). Elimination of extracellular bacteria by antibiotics in quantitative assays of bacterial ingestion and killing by phagocytes. J Immunol Methods.

[B24] Al-Robaiy S, Knauer J, Straubinger RK (2005). Borrelia burgdorferi organisms lacking plasmids 25 and 28-1 are internalized by human blood phagocytes at a rate identical to that of the wild-type strain. Infect Immun.

[B25] Fattorossi A, Nisini R, Pizzolo JG, D'Amelio R (1989). New, simple flow cytometry technique to discriminate between internalized and membrane-bound particles in phagocytosis. Cytometry.

[B26] Sambri V, Aldini R, Massaria F, Montagnani M, Casanova S, Cevenini R (1996). Uptake and killing of Lyme disease and relapsing fever borreliae in the perfused rat liver and by isolated Kupffer cells. Infect Immun.

[B27] Schwan TG, Burgdorfer W, Garon CF (1988). Changes in infectivity and plasmid profile of the Lyme disease spirochete, Borrelia burgdorferi, as a result of in vitro cultivation. Infect Immun.

[B28] Purser JE, Norris SJ (2000). Correlation between plasmid content and infectivity in Borrelia burgdorferi. Proc Natl Acad Sci U S A.

[B29] Georgilis K, Steere AC, Klempner MS (1991). Infectivity of Borrelia burgdorferi correlates with resistance to elimination by phagocytic cells. J Infect Dis.

[B30] Bisno AL, Stevens DL (1996). Streptococcal infections of skin and soft tissues. N Engl J Med.

[B31] Salazar JC, Pope CD, Sellati TJ, Feder HMJ, Kiely TG, Dardick KR, Buckman RL, Moore MW, Caimano MJ, Pope JG, Krause PJ, Radolf JD (2003). Coevolution of markers of innate and adaptive immunity in skin and peripheral blood of patients with erythema migrans. J Immunol.

[B32] Montgomery RR, Lusitani D, De Boisfleury CA, Malawista SE (2004). Tick saliva reduces adherence and area of human neutrophils. Infect Immun.

[B33] Anguita J, Samanta S, Revilla B, Suk K, Das S, Barthold SW, Fikrig E (2000). Borrelia burgdorferi gene expression in vivo and spirochete pathogenicity. Infect Immun.

[B34] Elias AF, Stewart PE, Grimm D, Caimano MJ, Eggers CH, Tilly K, Bono JL, Akins DR, Radolf JD, Schwan TG, Rosa P (2002). Clonal polymorphism of Borrelia burgdorferi strain B31 MI: implications for mutagenesis in an infectious strain background. Infect Immun.

[B35] Hartiala KT, Langlois L, Goldstein IM, Rosenbaum JT (1985). Endotoxin-induced selective dysfunction of rabbit polymorphonuclear leukocytes in response to endogenous chemotactic factors. Infect Immun.

[B36] Suhonen J, Hartiala K, Tuominen-Gustafsson H, Viljanen MK (2002). Sublethal concentrations of complement can effectively opsonize Borrelia burgdorferi. Scand J Immunol.

